# Clinical characteristics and risk factors of protein-losing enteropathy: a retrospective study

**DOI:** 10.3389/fimmu.2026.1760573

**Published:** 2026-05-08

**Authors:** Wen-Tao Tan, Zi-Teng Wang, Hui Su, Chun-Mei Guo, Wen-Bin Shen, Hong Liu

**Affiliations:** 1 Department of Gastroenterology, Beijing Shijitan Hospital, Capital Medical University, Beijing, China; 2 Liver Research Center, Beijing Friendship Hospital, Capital Medical University, Beijing, China; 3 Department of Gastroenterology, Beijing Ditan Hospital, Capital Medical University, Beijing, China; 4 Department of Lymphatic Surgery, Beijing Shijitan Hospital, Capital Medical University, Beijing, China

**Keywords:** autoimmunity, connective tissue disease, immunosuppressive therapy, mucosal barrier, protein-losing enteropathy, systemic lupus erythematosus

## Abstract

**Objective:**

Protein-losing enteropathy (PLE) is characterized by excessive gastrointestinal protein loss, yet systematic comparative studies across etiologies remain limited. This study aimed to characterize the confirmed PLE cohort in our center, with a focused comparison between connective tissue disease-associated PLE (CTD-PLE) and lymphatic drainage disorder-associated PLE (LDD-PLE), describe follow-up observations, and explore routine clinical indicators that may assist etiologic differentiation.

**Methods:**

This retrospective study included 146 patients admitted to Beijing Shijitan Hospital between January 2014 and December 2024 with PLE confirmed by 99mTc-HSA scintigraphy. Patients were classified as CTD-PLE or LDD-PLE according to the final clinical diagnosis. Clinical features, laboratory findings, and available follow-up data were analyzed, and logistic regression together with receiver operating characteristic (ROC) analyses were used to explore factors associated with CTD-PLE.

**Results:**

The cohort included 146 patients (median onset age 26 years; 30 CTD-PLE and 116 LDD-PLE). Edema (84.2%) and serous cavity effusions (76.7%) were the most frequent manifestations. Comparative analysis showed that CTD-PLE patients were older, predominantly female, and had more frequent thrombosis and higher D-dimer levels than LDD-PLE patients. CTD-PLE also showed distinct laboratory features, including higher total cholesterol, triglycerides, and globulin levels, whereas LDD-PLE was associated with lower lymphocyte counts and more frequent diarrhea. Multivariate analysis identified age at onset, Hb, and total cholesterol as independent predictors of CTD-PLE. The three-variable model showed good apparent discrimination in this single-center cohort (AUC = 0.890), while C3 showed the best single-variable discrimination. Among patients with available follow-up, 19/20 CTD-PLE patients receiving glucocorticoids plus immunosuppressants achieved symptom remission with a median ALB improvement of 9.5 (3.8, 19.6) g/L, whereas 23/43 surgically treated LDD-PLE patients achieved symptom remission with a median ALB improvement of 2.0 (-0.5, 5.8) g/L.

**Conclusion:**

PLE with different etiologies shows distinct clinical and laboratory patterns. Age at onset, Hb, and total cholesterol may assist preliminary etiologic differentiation, although the proposed model requires validation in independent cohorts. The observed lipid, coagulation, and correlation patterns, together with the follow-up findings, provide exploratory clues to distinct mechanisms and treatment trajectories.

## 1 Introduction

Protein-losing enteropathy (PLE) is a group of clinical syndromes characterized by nonselective hypoproteinemia caused by abnormal loss of plasma proteins through the gastrointestinal tract, typically presenting with concurrent decreases in serum albumin and immunoglobulins. The classic clinical manifestations of PLE include edema, diarrhea, steatorrhea, malnutrition, and an increased risk of infection (1, 2). PLE is not an independent disease entity but rather a shared clinical phenotype of multiple underlying disorders. Known etiologies include gastrointestinal diseases, lymphatic disorders, cardiovascular diseases, autoimmune diseases, and malignancies, among others (1). PLE is uncommon in the general population, and its incidence is closely related to the type of underlying disease. The diversity of etiologies suggests distinct pathophysiological mechanisms; a deeper understanding of these mechanisms is essential for clinical diagnosis and management.

According to the underlying pathophysiology, the causes of PLE can be broadly categorized into two major groups: intestinal mucosal injury (including erosive and non-erosive types) and impaired lymphatic drainage (1). Inflammatory bowel disease, peptic ulcers, infections, and malignancies may cause intestinal plasma protein loss by inducing erosive mucosal damage. In contrast, non-erosive mucosal injury is more commonly secondary to increased mucosal permeability due to autoimmune diseases (e.g., systemic lupus erythematosus [SLE], Sjögren’s syndrome), infections, or allergic reactions. Impaired lymphatic drainage may lead to pathological dilation and even rupture of intestinal lymphatic vessels, a condition termed intestinal lymphangiectasia (IL). Protein-rich lymph then leaks into the intestinal lumen, resulting in severe PLE. IL can be further classified as primary or secondary: primary IL is mainly due to congenital lymphatic developmental abnormalities, whereas secondary IL is caused by conditions that increase central venous pressure (e.g., cardiovascular diseases) or by extrinsic compression such as intra-abdominal tumors.

Moreover, a single underlying disease may involve multiple mechanisms described above (1, 3). This etiologic and mechanistic heterogeneity implies substantial variability in clinical features and prognosis among patients with PLE. Previous studies have shown that SLE-associated PLE often presents with multisystem involvement, disease-specific autoantibody positivity, and lipid metabolism abnormalities (4, 5), whereas PLE related to impaired lymphatic drainage may be dominated by diarrhea/steatorrhea and marked lymphopenia (6, 7). In addition, SLE-associated PLE often responds to glucocorticoids with or without immunosuppressants (4, 8–11). Other connective tissue diseases have also been reported to share mechanisms similar to SLE and to respond to immunotherapy (12, 13). However, existing research has largely focused on single etiologies, and systematic comparisons of clinical differences across etiologic categories (particularly autoimmune diseases vs. lymphatic drainage disorders) remain limited. This knowledge gap hampers a deeper understanding of the clinical heterogeneity of PLE and poses challenges to precise diagnosis and tailored treatment.

Therefore, this study retrospectively analyzed the clinical data of patients diagnosed with PLE in our hospital. We first summarized the overall characteristics of the scintigraphy-confirmed cohort and then performed a focused comparison between CTD-PLE and LDD-PLE to identify clinical indicators that may aid etiologic discrimination and targeted management. By combining overall cohort description with between-group analysis, we aimed to support earlier etiologic recognition and provide a clinical basis for future mechanistic studies.

## 2 Materials and methods

### 2.1 Study population

This study included patients with PLE hospitalized at Beijing Shijitan Hospital, Capital Medical University, between January 2014 and December 2024. A total of 205 suspected PLE records were retrieved from the hospital information system using the search term “protein-losing enteropathy”. After first-round screening, 53 records were excluded, including 4 with alternative primary etiologies outside the prespecified analytic framework and 49 considered mixed etiologies. The remaining 152 records underwent detailed etiologic adjudication. Six additional cases were excluded, including 5 patients in whom hypoalbuminemia was judged primarily attributable to lupus nephritis rather than intestinal protein loss and 1 patient whose final clinical diagnosis was non-Hodgkin lymphoma. Thus, 146 patients were included in the final analytic cohort (60 male [41.1%] and 86 female [58.9%]); the overall selection pathway is summarized in **Supplementary Figure S1**.

### 2.2 Inclusion and exclusion criteria

Patients were eligible for inclusion if PLE was confirmed by positive technetium-99m-labeled human serum albumin scintigraphy (99mTc-HSA) and they had clinical hypoproteinemia (serum albumin <35 g/L), with or without typical PLE manifestations such as edema, diarrhea, or serous cavity effusions; moreover, patients were required to have core baseline medical records, including demographic information, presenting symptoms, and essential laboratory evaluation. Conversely, patients were excluded if they had concomitant malignancy, definite renal protein loss (24-h urinary protein >0.5 g/24 h), cirrhosis or other conditions with clearly reduced hepatic protein synthesis, severe malnutrition clearly attributable to non-PLE causes (e.g., prolonged fasting, anorexia nervosa, or overt malabsorption), or mixed mechanisms that precluded adjudication to a dominant CTD-PLE or LDD-PLE category.

### 2.3 Data collection and follow-up

Baseline data were retrospectively collected at admission, including sex, age at admission, age at onset, and primary diagnosis. Laboratory parameters included white blood cells (WBC), neutrophils (NE), lymphocytes (LY), hemoglobin (Hb), platelets (PLT), albumin (ALB), globulin (GLB), total cholesterol (TC), triglycerides (TG), low-density lipoprotein cholesterol (LDL-C), high-density lipoprotein cholesterol (HDL-C), calcium (Ca), C-reactive protein (CRP), erythrocyte sedimentation rate (ESR), D-dimer, immunoglobulin G (IgG), immunoglobulin A (IgA), immunoglobulin M (IgM), complement 3 (C3), complement 4 (C4), and antinuclear antibody (ANA). Clinical symptoms (edema, abdominal pain, abdominal distension, diarrhea, vomiting, fever, serous effusions, and thrombosis) were also recorded. Laboratory tests were performed in the hospital clinical laboratory according to routine institutional procedures. Baseline data were defined as clinical and laboratory results obtained at the first admission before being influenced by in-hospital treatment. Not every laboratory variable was available for every patient, and the available sample size for each variable is reported in the tables where applicable. Objective fat malabsorption assessments (e.g., 72-h fecal fat quantification or Sudan staining), detailed stool characteristics, fibrinogen, provoking factors for thrombosis, and antiphospholipid antibody profiles were not uniformly available in the retrospective records and therefore were not included in the predefined analyses.

Follow-up information was collected with a censoring date of December 31, 2024. Treatment regimens, follow-up duration, symptom remission, and follow-up laboratory values (including changes in ALB and GLB) were recorded.

### 2.4 Scintigraphy protocol and image interpretation

Imaging was performed using a Philips Forte dual-head SPECT system equipped with a low-energy high-resolution collimator. The tracer was technetium-99m–labeled human serum albumin (99mTc-HSA), administered at 740 MBq (1–2 mL) for adults, while pediatric doses were calculated using the Webster formula based on body weight. Static anterior and posterior abdominal images were acquired at 10 min, 1 h, 2 h, 3 h, 6 h, and 24 h after intravenous injection, using a 512×512 matrix, a 141 keV energy peak with a 20% window, and a zoom factor of 1. Acquisition time was 300 s for the 10 min/1 h/2 h/3 h/6 h images and 600 s for the 24 h images.

Images were jointly interpreted by at least two nuclear medicine physicians, and discrepancies were resolved by discussion. Image interpretation included assessment of intestinal visualization (presence/absence, time of initial appearance, location, extent, and temporal changes in radioactivity), abnormal radioactivity distribution within the abdominal cavity, and other abnormal tracer distributions.

### 2.5 Diagnostic criteria

Because no unified international diagnostic criteria exist for PLE, diagnosis in this study was based on hypoproteinemia (serum albumin <35 g/L) together with intestinal protein leakage confirmed by positive 99mTc-HSA scintigraphy. Primary diseases were diagnosed according to internationally accepted criteria for each condition.

### 2.6 Etiologic grouping

In keeping with the final clinical diagnosis documented for each patient and the predominant pathophysiologic pattern, the final analytic cohort was stratified into two prespecified groups: connective tissue disease-associated PLE (CTD-PLE) and lymphatic drainage disorder-associated PLE (LDD-PLE). CTD-PLE included conditions such as systemic lupus erythematosus, Sjögren’s syndrome, systemic sclerosis, rheumatoid arthritis, and undifferentiated connective tissue disease, all diagnosed according to recognized classification criteria (e.g., the American College of Rheumatology/European Alliance of Associations for Rheumatology [ACR/EULAR] criteria), in which PLE was mainly attributed to non-erosive mucosal permeability abnormalities. LDD-PLE comprised cases due to primary or secondary intestinal lymphangiectasia, cardiac-related elevated central venous pressure, and other mechanisms leading to lymph leakage into the intestinal lumen. In this retrospective study, etiologic grouping was assigned according to the final clinical diagnosis recorded in the medical record, supported by the available clinical, laboratory, and imaging findings. Cases considered mixed etiology or lacking a dominant final clinical diagnosis were excluded from the analytic cohort.

### 2.7 Study outcomes

The study objectives were to summarize the baseline characteristics of the confirmed PLE cohort; perform focused comparisons between CTD-PLE and LDD-PLE and identify independent predictors of CTD-PLE, including diagnostic cutoffs and apparent performance; assess correlations between baseline ALB and other baseline indicators within each subgroup; and describe follow-up characteristics and post-treatment outcomes among patients with available follow-up data, while exploring baseline factors associated with outcomes within the LDD-PLE subgroup.

### 2.8 Statistical analysis

SPSS 27.0 was used. Continuous variables were tested for normality; normally distributed data were reported as mean ± standard deviation, and non-normally distributed data as median (interquartile range). Categorical variables were reported as n (%). Between-group comparisons used independent-samples t tests or Mann-Whitney U tests for continuous variables, and chi-square tests or Fisher’s exact tests for categorical variables. Spearman or Pearson correlation and linear regression were used to evaluate associations between baseline ALB and other variables within each subgroup. Univariate logistic regression was used to screen potential predictors distinguishing PLE etiology; variables were selected for multivariable logistic regression based on statistical significance, clinical relevance, subgroup size, and data availability. Receiver operating characteristic (ROC) curves and area under the curve (AUC) were used to evaluate diagnostic performance. Within the LDD-PLE subgroup, univariate logistic regression explored baseline factors associated with symptom remission; for continuous prognostic outcomes, Spearman correlation screening was followed by multivariable linear regression (with backward elimination) including baseline ALB. Collinearity was assessed. Owing to the retrospective design, data completeness varied across variables; analyses were performed on an available-case basis, and the number of patients with available data is reported in the tables where applicable. Because most comparisons were exploratory, no formal adjustment for multiple testing was applied, and findings from secondary analyses should be interpreted cautiously. The ROC performance reported for the combined model represents apparent performance in this single-center cohort, as no internal resampling or external validation dataset was available. Exploratory *post hoc* sensitivity analyses additionally examined between-group differences in available baseline D-dimer, the association of TC with CTD-PLE after adjustment for baseline ALB, and the apparent performance of a C3-augmented diagnostic model. All tests were two-sided, with P < 0.05 considered statistically significant.

## 3 Results

### 3.1 Overall cohort characteristics

#### 3.1.1 Etiology and demographics

A total of 146 patients with PLE diagnosed by 99mTc-HSA scintigraphy and hospitalized between January 2014 and December 2024 were included; the screening and cohort assembly process is summarized in **Supplementary Figure S1**. The cohort comprised 60 men (41.1%) and 86 women (58.9%). Median age at admission was 32 (12, 51) years, and median age at onset was 26 (6, 47) years. Underlying etiologies were heterogeneous, including primary lymphatic disorders (n=104), SLE (n=22), undifferentiated connective tissue disease (n=4), constrictive pericarditis (n=4), congenital heart disease (n=4), restrictive cardiomyopathy (n=2), chronic heart failure (n=2), overlap syndrome (n=1), Sjögren’s syndrome (n=1), systemic sclerosis (n=1), and rheumatoid arthritis (n=1).

#### 3.1.2 Clinical manifestations

The most common symptoms were edema (84.2%, 123/146) and serous cavity effusions (76.7%, 112/146), including ascites (58.2%, 85/146), pleural effusion (47.9%, 70/146), and pericardial effusion (23.3%, 34/146). Other symptoms included diarrhea (51.4%, 75/146), abdominal distension (30.1%, 44/146), abdominal pain (13.7%, 20/146), vomiting (11.0%, 16/146), fever (4.8%, 7/146), and thrombotic events (6.8%, 10/146).

#### 3.1.3 Key laboratory findings

Severe hypoalbuminemia was the most prominent abnormality (mean ALB 23.5 ± 6.0 g/L). Other abnormalities included hypoglobulinemia (GLB 17.8 [14.8, 24.2] g/L), lymphopenia (LY 1.1 [0.7, 1.6] ×10^9/L), hypocalcemia (Ca 1.96 [1.83, 2.07] mmol/L), and reduced immunoglobulin levels: IgG 4.80 (3.04, 7.81) g/L, IgA 0.88 (0.56, 1.49) g/L, and IgM 0.64 (0.41, 1.08) g/L. D-dimer was measured in 112 patients (76.7%): 268.0 (117.5, 519.5) µg/L. ANA was positive in 37 patients (25.3%) (**Table 1**).

**Table 1:** Baseline clinical characteristics of patients with protein-losing enteropathy (PLE).

Variable	Total (N = 146)
Demographics	
Male, n (%)	60 (41.1%)
Age at admission (yr)	32 (12, 51)
Age at onset (yr)	26 (6, 47)
Clinical symptoms, n (%)	
Edema	123 (84.2%)
Abdominal pain	20 (13.7%)
Abdominal distension	44 (30.1%)
Diarrhea	75 (51.4%)
Vomiting	16 (11.0%)
Fever	7 (4.8%)
Serous cavity effusions	112 (76.7%)
Pleural effusion	70 (47.9%)
Ascites	85 (58.2%)
Pericardial effusion	34 (23.3%)
Thrombosis	10 (6.8%)
Laboratory findings	
Hb (g/L)	128.4 ± 21.8
ALB (g/L)	23.5 ± 6.0
WBC (×10^9/L)	6.3 (5.0, 8.2)
NE (×10^9/L)	4.4 (3.5, 6.1)
LY (×10^9/L)	1.1 (0.7, 1.6)
PLT (×10^9/L)	285 (211, 374)
GLB (g/L)	17.8 (14.8, 24.2)
TC (mmol/L)	3.8 (3.3, 4.9)
TG (mmol/L)	1.1 (0.8, 1.8)
LDL-C (mmol/L)	2.5 (2.0, 3.2)
HDL-C (mmol/L)	0.9 (0.7, 1.0)
Ca (mmol/L)	1.96 (1.83, 2.07)
IgG (g/L)	4.80 (3.04, 7.81)
IgA (g/L)	0.88 (0.56, 1.49)
IgM (g/L)	0.64 (0.41, 1.08)
C3 (g/L)	0.99 (0.72, 1.16)
C4 (g/L)	0.22 (0.14, 0.28)
CRP (mg/L) (n=90)	3.0 (0.6, 8.2)
ESR (mm/h) (n=85)	11 (6, 44)
D-dimer (μg/L) (n=112)	268.0 (117.5, 519.5)
ANA positive, n (%)	37 (25.3%)

### 3.2 Between-group comparison of PLE with different etiologies

#### 3.2.1 Grouping and demographics

Patients were divided into a CTD-PLE group (n=30) and an LDD-PLE group (n=116). Significant differences were observed across demographics, clinical features, and laboratory findings (**Table 2**). Compared with the LDD-PLE group, the CTD-PLE group had older age at admission (median 42 vs. 28 years, P = 0.002) and older age at onset (median 36 vs. 17 years, P<0.001), and a higher proportion of females (86.7% vs. 51.7%, P<0.001).

**Table 2:** Comparison of baseline clinical characteristics in PLE patients with different etiologies.

Variable	CTD-PLE group (n=30)	LDD-PLE group (n=116)	P value
Demographics			
Age at admission (yr)	42 (30, 52)	28 (9, 51)	**0.002**
Age at onset (yr)	36 (26, 50)	17 (3, 43)	**<0.001**
Male, n (%)	4 (13.3%)	56 (48.3%)	**<0.001**
Clinical symptoms, n (%)			
Edema	25 (83.3%)	98 (84.5%)	1
Abdominal pain	4 (13.3%)	16 (13.8%)	1
Abdominal distension	12 (40.0%)	32 (27.6%)	0.187
Diarrhea	9 (30.0%)	66 (56.9%)	**0.009**
Vomiting	5 (16.7%)	11 (9.5%)	0.323
Fever	3 (10.0%)	4 (3.4%)	0.153
Serous cavity effusions	26 (86.7%)	86 (74.1%)	0.148
Pleural effusion	17 (56.7%)	53 (45.7%)	0.283
Ascites	20 (66.7%)	65 (56.0%)	0.293
Pericardial effusion	10 (33.3%)	24 (20.7%)	0.144
Thrombosis	7 (23.3%)	3 (2.6%)	**<0.001**
Laboratory indicators			
Hb (g/L)	113.2 ± 21.6	132.3 ± 20.1	**<0.001**
ALB (g/L)	22.4 ± 7.9	23.8 ± 5.5	0.346
WBC (×10^9/L)	7.2 (5.7, 9.8)	6.3 (5.0, 8.1)	0.444
NE (×10^9/L)	4.7 (3.3, 7.4)	4.4 (3.5, 6.0)	0.947
LY (×10^9/L)	1.4 (1.1, 1.9)	0.9 (0.7, 1.5)	**0.019**
PLT (×10^9/L)	226 (161, 321)	304 (233, 396)	**<0.001**
GLB (g/L)	24.3 (19.5, 27.8)	16.8 (14.4, 21.5)	**<0.001**
TC (mmol/L)	6.5 (3.8, 8.0)	3.7 (3.2, 4.9)	**<0.001**
TG (mmol/L)	1.9 (1.3, 3.6)	1.1 (0.7, 1.4)	**<0.001**
LDL-C (mmol/L)	3.7 (2.5, 5.2)	2.3 (1.9, 2.8)	**<0.001**
HDL-C (mmol/L)	0.8 (0.6, 1.0)	0.9 (0.7, 1.0)	0.231
Ca (mmol/L)	1.96 (1.79, 2.11)	1.96 (1.84, 2.07)	0.953
IgG (g/L)	8.00 (4.91, 12.90)	4.19 (2.78, 5.96)	**<0.001**
IgA (g/L)	1.93 (1.25, 2.59)	0.79 (0.52, 1.24)	**<0.001**
IgM (g/L)	0.91 (0.63, 1.38)	0.57 (0.39, 1.00)	**0.001**
C3 (g/L)	0.66 (0.57, 0.72)	1.03 (0.89, 1.18)	**<0.001**
C4 (g/L)	0.14 (0.10, 0.19)	0.24 (0.19, 0.31)	**<0.001**
D-dimer (μg/L)	476.0 (309.0, 794.0)	167.0 (103.5, 382.0)	<0.001
ANA positive, n (%)	24 (80.0%)	13 (11.2%)	**<0.001**

Bold values indicate statistically significant differences (P < 0.05).

#### 3.2.2 Clinical manifestations

Thrombotic events were significantly more frequent in the CTD-PLE group (23.3% vs. 2.6%, P<0.001), whereas diarrhea was more common in the LDD-PLE group (56.9% vs. 30.0%, P = 0.009). No significant differences were found for edema, abdominal pain, abdominal distension, vomiting, fever, or serous cavity effusions (pleural, ascitic, or pericardial) (all P>0.05).

#### 3.2.3 Laboratory findings

The CTD-PLE group had significantly higher GLB, TC, TG, LDL-C, and IgG/IgA/IgM levels, and a higher ANA positivity rate; C3 and C4 levels were significantly lower (all P ≤ 0.001). The LDD-PLE group had higher Hb and PLT (P<0.001) and lower LY (P = 0.019). WBC, ALB, Ca, and HDL-C did not differ significantly (all P>0.05). Baseline D-dimer, available in 112 patients, was higher in CTD-PLE than in LDD-PLE [476.0 (309.0, 794.0) vs. 167.0 (103.5, 382.0) μg/L, P<0.001].

#### 3.2.4 Subgroup analyses

To further explore the internal consistency of the grouping strategy (CTD-PLE vs. LDD-PLE), subgroup analyses were performed within CTD-PLE (SLE, n=22 vs. non-SLE, n=8) and within LDD-PLE (primary lymphatic disorders, n=104 vs. cardiovascular diseases, n=12). Most clinical manifestations and laboratory indicators did not differ significantly within these subgroups (all P>0.05). Only Hb differed significantly between SLE and non-SLE within CTD-PLE (126.5 ± 19.4 vs. 108.4 ± 20.6 g/L, P = 0.045).

#### 3.2.5 Univariate and multivariate logistic regression

Univariate analyses showed that female sex, age at onset, diarrhea, thrombotic events, and laboratory parameters (Hb, PLT, GLB, TC, TG, LDL-C, IgG, IgA, C3, C4, and ANA positivity) were significantly associated with CTD-PLE (all P<0.05). Considering subgroup size, data availability, and clinical practicality, age at onset, Hb, and TC were entered into a multivariable logistic regression model. Age at onset (OR = 1.030, 95% CI: 1.001-1.059, P = 0.045), TC (OR = 2.186, 95% CI: 1.570-3.045, P<0.001), and Hb (OR = 0.934, 95% CI: 0.901-0.965, P<0.001) were identified as independent predictors for distinguishing CTD-PLE from LDD-PLE (**Table 3**). In an exploratory sensitivity analysis adjusting TC for baseline ALB, TC remained independently associated with CTD-PLE (OR = 2.112, 95% CI: 1.570-2.841, P<0.001), whereas baseline ALB was not significant (P = 0.339).

**Table 3:** Univariate and multivariate analysis of predictors for CTD-PLE.

Variable	Univariate		Multivariate	
	P value	*OR(95% CI)*	P value	*OR(95% CI)*
Female	0.002	6.067 (1.992–18.480)	–	–
Age at onset (yr)	0.001	1.034 (1.013–1.055)	0.045	1.030 (1.001 - 1.059)
Diarrhea	0.011	0.325 (0.137–0.770)	–	–
Thrombosis	<0.001	11.464 (2.757–47.659)	–	–
Hb	<0.001	0.958 (0.937–0.978)	<0.001	0.934 (0.901 - 0.965)
PLT	0.005	0.994 (0.980–0.998)	–	–
GLB	<0.001	1.144 (1.071–1.223)	–	–
TC	<0.001	2.001 (1.532–2.612)	<0.001	2.186 (1.570 - 3.045)
TG	<0.001	1.808 (1.316–2.486)	–	–
LDL-C	<0.001	1.988 (1.458–2.710)	–	–
IgG	<0.001	1.153 (1.063–1.250)	–	–
IgA	<0.001	2.690 (1.701–4.252)	–	–
C3	<0.001	0.001 (0.000–0.003)	–	–
C4	<0.001	0.001 (0.000–0.001)	–	–
ANA positive	<0.001	31.692 (10.931–91.886)	–	–

#### 3.2.6 ROC analysis

**Figure 1:**
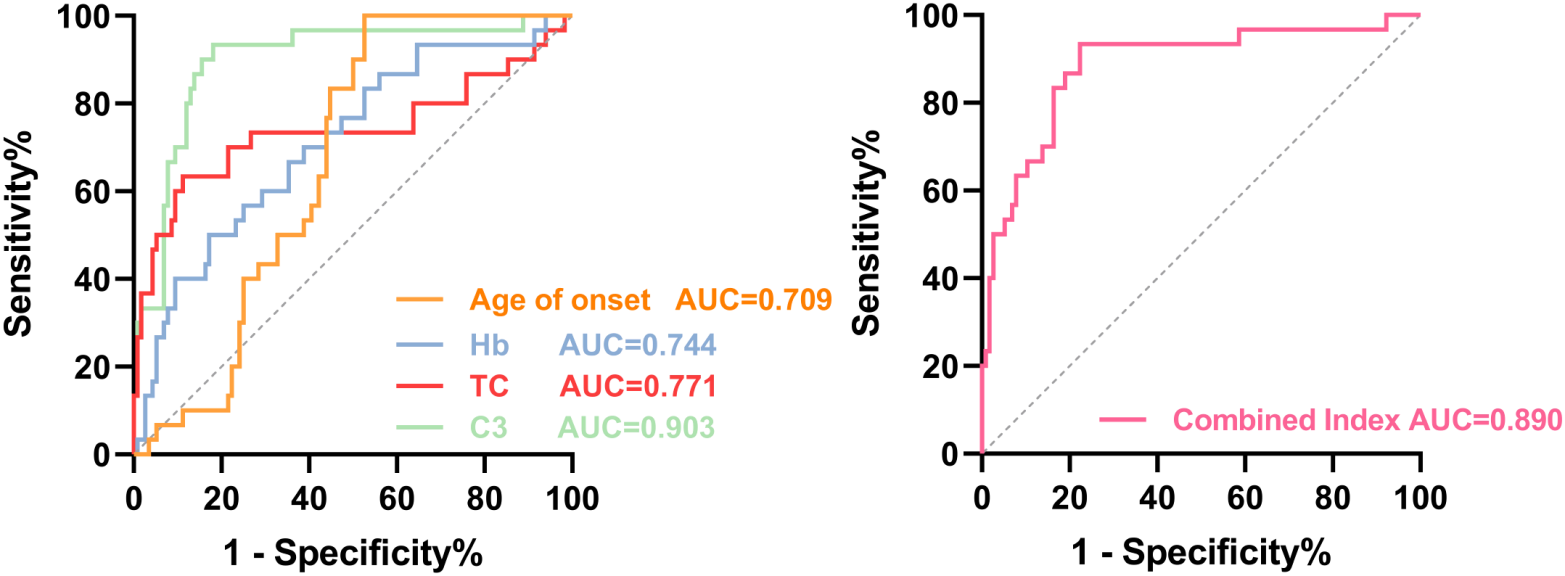
ROC curve analysis of single indicators and combined models for distinguishing the etiology of PLE.

Among individual indicators, C3 showed the best apparent diagnostic performance for CTD-PLE (AUC = 0.903), followed by TC (AUC = 0.771), Hb (AUC = 0.744), and age at onset (AUC = 0.709) (**Figure 1**). A combined model using clinically accessible variables (age at onset, Hb, and TC) showed an apparent AUC of 0.890 within this derivation cohort (**Figure 1**), with sensitivity of 90.0% and specificity of 84.5%. The optimal single-variable thresholds for TC and Hb were 5.28 mmol/L and 123.5 g/L, respectively; within the combined model, stricter operational thresholds were used (TC 6.3 mmol/L and Hb 100 g/L). An available-case *post hoc* sensitivity model adding C3 to age at onset, Hb, and TC achieved an apparent AUC of 0.964 in the derivation cohort.

### 3.3 Correlation analyses between baseline ALB and other indicators

#### 3.3.1 CTD-PLE subgroup

**Figure 2:**
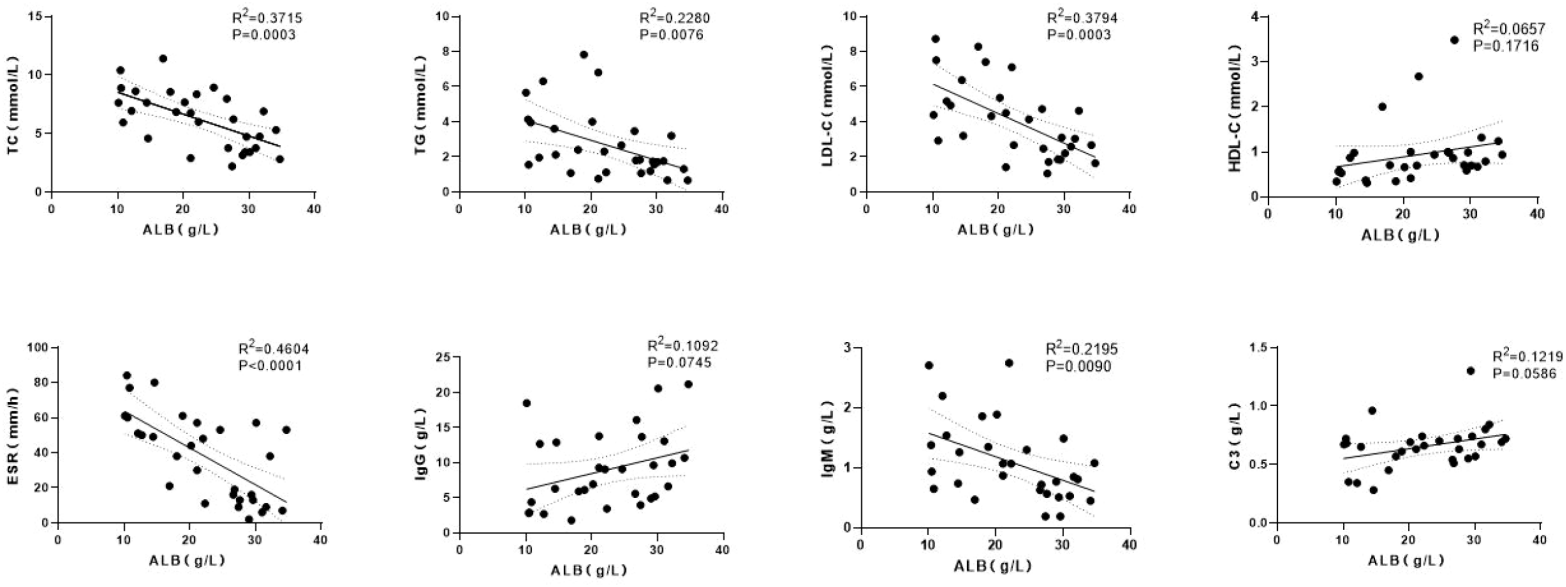
Correlation analysis between baseline ALB and selected clinical indicators in the CTD-PLE group.

In the CTD-PLE group, Spearman correlation and linear regression analyses showed significant negative correlations between ALB and ESR (ρ=-0.654, P<0.001), LDL-C (ρ=-0.625, P<0.001), TC (ρ=-0.603, P<0.001), TG (ρ=-0.545, P = 0.002), and IgM (ρ=-0.469, P = 0.009). Significant positive correlations were observed between ALB and HDL-C (ρ=0.470, P = 0.009) and IgG (ρ=0.383, P = 0.037). The positive correlation between ALB and C3 approached statistical significance (ρ=0.350, P = 0.059), while ALB showed no significant correlation with WBC, CRP, or IgA. Linear regression further supported significant negative linear relationships between ALB and ESR (R²=0.460, P<0.001), LDL-C (R²=0.379, P<0.001), TC (R²=0.372, P<0.001), TG (R²=0.228, P = 0.008), and IgM (R²=0.220, P = 0.009). Linear relationships with HDL-C (R²=0.066, P = 0.172), IgG (R²=0.109, P = 0.075), and C3 (R²=0.122, P = 0.059) were not statistically significant (**Figure 2**).

#### 3.3.2 LDD-PLE subgroup

**Figure 3:**
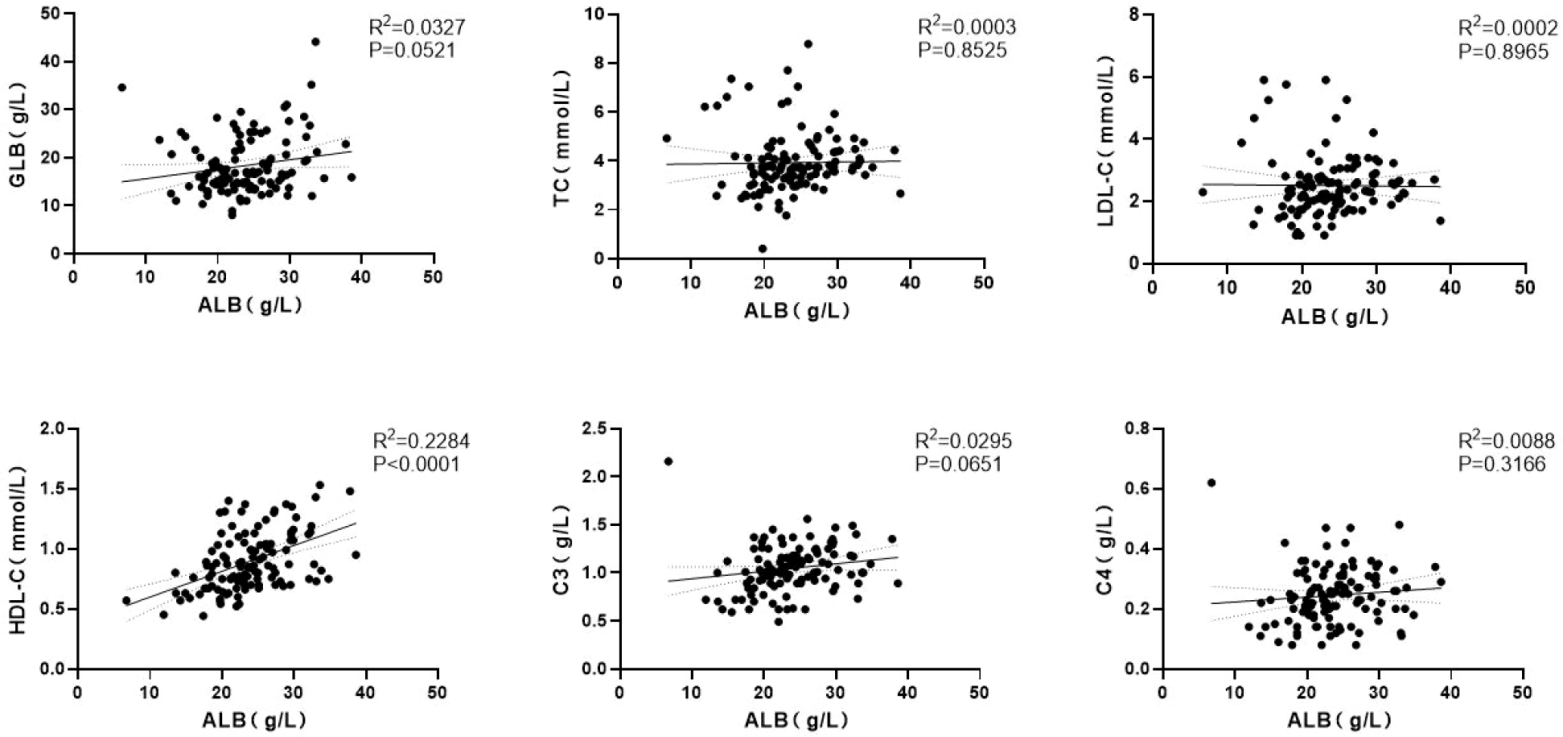
Correlation analysis between baseline ALB and selected clinical indicators in the LDD-PLE group.

In the LDD-PLE group, baseline ALB was positively correlated with GLB (ρ=0.197, P = 0.034), TC (ρ=0.197, P = 0.035), LDL-C (ρ=0.183, P = 0.049), HDL-C (ρ=0.473, P<0.001), C3 (ρ=0.298, P = 0.001), and C4 (ρ=0.207, P = 0.026). Linear regression demonstrated a significant linear relationship only between ALB and HDL-C (R²=0.228, P<0.001). No significant linear relationships were observed for ALB with GLB (R²=0.033, P = 0.052), TC (R²<0.001, P = 0.853), LDL-C (R²<0.001, P = 0.897), C3 (R²=0.030, P = 0.065), or C4 (R²=0.009, P = 0.317) (**Figure 3**).

### 3.4 Follow-up and exploratory prognostic analysis

#### 3.4.1 Descriptive follow-up observations

**Figure 4:**
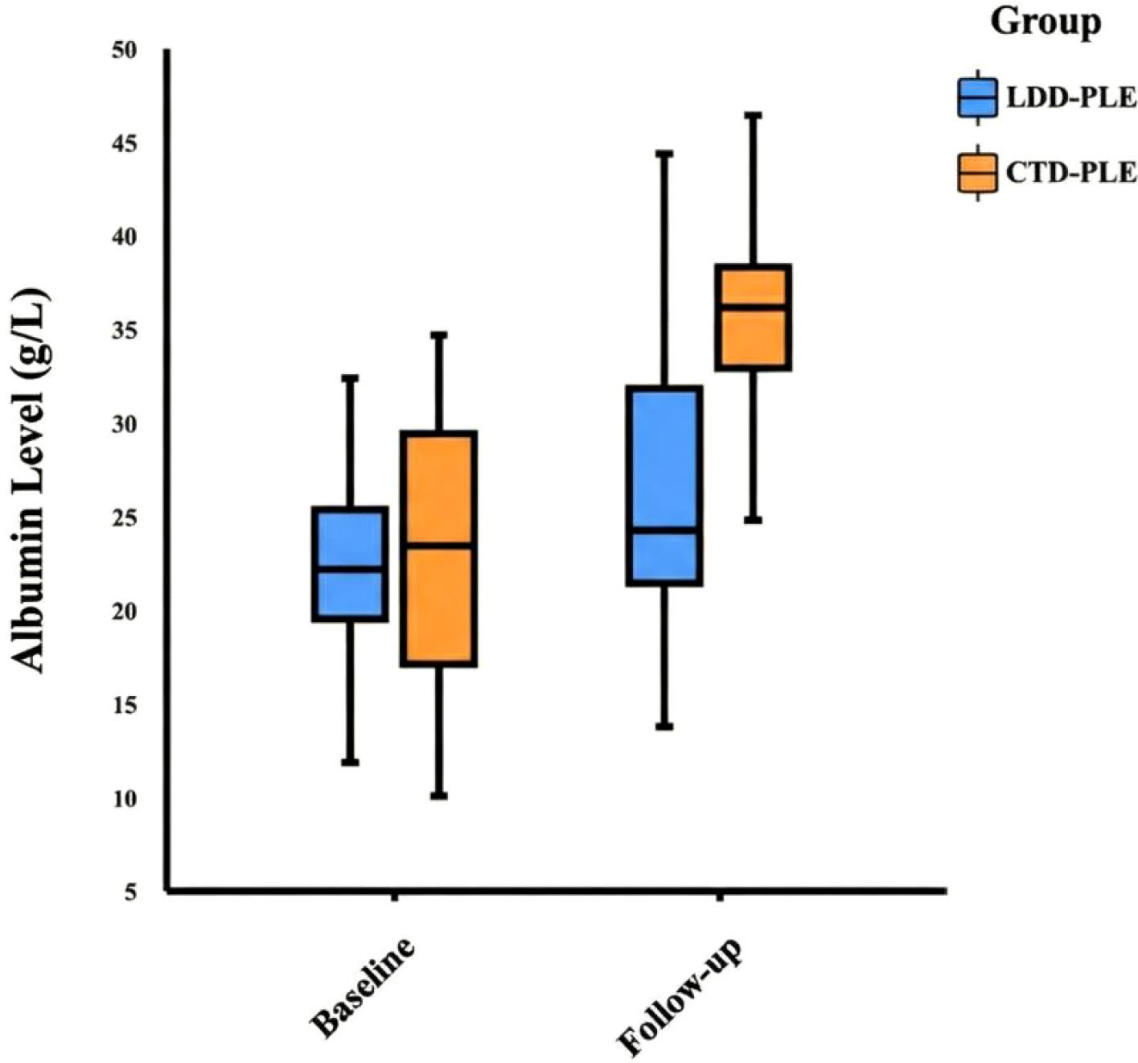
Box plot showing changes in albumin levels before and after follow-up.

A total of 63 patients with available follow-up data were included: 20 in the CTD-PLE group and 43 in the LDD-PLE group. All CTD-PLE patients with follow-up received glucocorticoids combined with immunosuppressants, whereas all LDD-PLE patients in the follow-up cohort had lymphatic disorders and underwent surgical treatment. In the CTD-PLE subgroup, median follow-up duration was 11 (5, 17) months, 19/20 (95.0%) achieved symptom remission, follow-up ALB was 34.8 ± 7.4 g/L, follow-up GLB was 25.7 (21.3, 30.1) g/L, and median ALB improvement was 9.5 (3.8, 19.6) g/L. In the LDD-PLE subgroup, median follow-up duration was 18 (8, 46) months, 23/43 (53.5%) achieved symptom remission, follow-up ALB was 26.1 ± 7.6 g/L, follow-up GLB was 17.5 (15.2, 21.8) g/L, and median ALB improvement was 2.0 (-0.5, 5.8) g/L. Owing to fundamentally different treatment modalities and unequal follow-up duration, these outcomes are reported descriptively (**Table 4**; **Figure 4**).

**Table 4:** Baseline characteristics and descriptive outcomes in the follow-up cohort.

Variable	Total (n=63)	CTD-PLE group (n=20)	LDD-PLE group (n=43)
Demographics			
Age at admission (yr)	32 (13, 52)	42 (30, 52)	23 (10, 51)
Age at onset (yr)	26 (10, 47)	35 (26, 50)	14 (3, 36)
Male, n (%)	27 (42.9%)	3 (15.0%)	24 (55.8%)
Follow-up outcomes			
Symptom remission, n (%)	42 (66.7%)	19 (95.0%)	23 (53.5%)
Follow-up duration (months)	13 (7, 34)	11 (5, 17)	18 (8, 46)
Follow-up ALB (g/L)	28.8 ± 8.5	34.8 ± 7.4	26.1 ± 7.6
Follow-up GLB (g/L)	20.1 (16.2, 24.9)	25.7 (21.3, 30.1)	17.5 (15.2, 21.8)
Improvement in ALB (g/L)	3.6 (0.7, 9.9)	9.5 (3.8, 19.6)	2.0 (-0.5, 5.8)
Improvement in GLB (g/L)	1.0 (-2.8, 3.6)	-0.6 (-3.8, 3.4)	1.5 (-1.6, 4.0)
Baseline indicators			
Hb (g/L)	125.0 ± 23.6	112.7 ± 22.4	130.7 ± 22.2
ALB (g/L)	22.7 ± 5.8	22.9 ± 7.8	22.6 ± 4.7
WBC (×10^9/L)	6.7 (5.6, 7.9)	7.2 (6.0, 9.5)	6.5 (5.4, 7.6)
NE (×10^9/L)	4.8 (3.8, 6.1)	4.9 (4.0, 7.2)	4.7 (3.7, 6.0)
LY (×10^9/L)	1.1 (0.7, 1.5)	1.4 (0.9, 1.8)	0.9 (0.6, 1.4)
PLT (×10^9/L)	304 (219, 412)	220 (131, 339)	343 (244, 424)
GLB (g/L)	18.5 (15.0, 24.4)	25.9 (20.5, 30.2)	16.4 (14.4, 19.8)
TC (mmol/L)	3.8 (3.3, 6.1)	6.5 (4.2, 7.8)	3.5 (3.1, 4.2)
TG (mmol/L)	1.2 (0.7, 1.8)	1.9 (1.6, 3.1)	0.9 (0.7, 1.5)
LDL-C (mmol/L)	2.4 (2.0, 3.3)	3.7 (2.5, 5.3)	2.2 (2.0, 2.6)
HDL-C (mmol/L)	0.9 (0.7, 1.0)	0.7 (0.6, 1.0)	0.9 (0.7, 1.0)
IgG (g/L)	5.19 (3.41, 7.88)	9.38 (6.03, 13.40)	3.97 (2.82, 5.79)
IgA (g/L)	1.16 (0.64, 1.89)	2.04 (1.53, 2.80)	0.74 (0.53, 1.21)
IgM (g/L)	0.57 (0.43, 1.13)	1.08 (0.60, 1.68)	0.52 (0.38, 0.93)
C3 (g/L)	0.88 ± 0.26	0.66 ± 0.21	0.98 ± 0.22
C4 (g/L)	0.21 (0.14, 0.27)	0.12 (0.09, 0.19)	0.22 (0.19, 0.27)
D-dimer (μg/L)	318.0 (129.0, 633.0)	539.0 (309.0, 818.0)	150.5 (95.8, 382.5)
ANA positive, n (%)	24 (38.1%)	17 (85.0%)	7 (16.3%)

#### 3.4.2 Exploratory prognostic factors in the LDD-PLE subgroup

To explore baseline factors potentially associated with outcomes in the LDD-PLE subgroup, analyses were performed for both symptom remission and ALB improvement. Among baseline laboratory variables, none was significantly associated with symptom remission (all P>0.05). Spearman correlation analyses of ALB improvement showed significant negative correlations with WBC (ρ=-0.345, P = 0.022), IgA (ρ=-0.399, P = 0.007), and IgM (ρ=-0.355, P = 0.018). A multivariable linear regression model was then constructed using IgA (the strongest correlate) and baseline ALB as an adjustment covariate. The model was statistically significant (F = 5.059, P = 0.011) and explained approximately 16.2% of the variance in ALB improvement (adjusted R²=0.162). Baseline IgA was an independent negative predictor of ALB improvement: for each 1 g/L increase in IgA, mean ALB improvement decreased by 4.31 g/L (B=-4.308, 95% CI: -7.191 to -1.424, P = 0.004). Baseline ALB was not an independent predictor (B=-0.157, 95% CI: -0.561 to 0.246, P = 0.435). Given the limited sample size and retrospective design, these subgroup prognostic analyses should be considered exploratory.

## 4 Discussion

In this study, we retrospectively analyzed the clinical characteristics, laboratory profiles, and available follow-up observations of 146 patients with PLE confirmed by 99mTc-HSA scintigraphy at Beijing Shijitan Hospital over the past decade. Epidemiologic data on PLE remain limited, and prior studies have largely focused on PLE associated with a single etiology. For example, the prevalence of PLE among patients with SLE has been reported to be approximately 1-3% (4, 8, 9), whereas the incidence of PLE after the Fontan procedure can reach 5-18% (2, 3), underscoring that PLE frequency depends strongly on the underlying disease. However, systematic comparisons of clinical differences across etiologic categories are scarce. To our knowledge, this is the first retrospective cohort study in China to compare PLE attributable to different etiologic categories within a scintigraphy-confirmed cohort. In our cohort, PLE predominantly affected adolescents and young to middle-aged adults (median age at onset 26 years), with a slight female predominance (58.9%). Severe hypoproteinemia and fluid retention were the most common features, with most patients presenting with peripheral edema (84.2%) and serous cavity effusions (76.7%). Gastrointestinal symptoms occurred in a subset, most commonly diarrhea (51.4%), abdominal distension (30.1%), and abdominal pain (13.7%), consistent with prior reports (14). Laboratory evaluations further indicated that PLE is commonly accompanied by hypoglobulinemia, reductions in multiple immunoglobulins (IgG, IgA, IgM), lymphopenia, and hypocalcemia, consistent with nonselective plasma protein loss and lymphocyte depletion (1, 15).

Given the broad disease spectrum, and in keeping with the final clinical diagnosis documented for each patient, we stratified the final analytic cohort into the two prespecified groups of CTD-PLE and LDD-PLE according to the predominant underlying mechanism. Because the final analytic cohort was restricted to these two categories, the inferential findings of this study primarily inform differentiation between CTD-PLE and LDD-PLE rather than the entire etiologic spectrum of PLE. CTD-PLE is primarily driven by autoimmune-mediated abnormalities in intestinal mucosal permeability, whereas LDD-PLE is predominantly related to structural factors such as cardiac dysfunction or impaired lymphatic drainage. This categorization aligns with existing literature (1). Marked demographic differences were observed: CTD-PLE tended to occur at older ages and predominantly in women, likely reflecting the female predominance of connective tissue diseases. Symptom profiles differed modestly: while edema, serous effusions, and common gastrointestinal symptoms (abdominal pain and distension) were similar, diarrhea was less frequent in CTD-PLE than in LDD-PLE. However, objective assessments of fat malabsorption (e.g., 72-h fecal fat quantification or Sudan staining) and detailed stool characteristics were not systematically captured, so the combination of lower diarrhea frequency and higher lipid levels in CTD-PLE should not be interpreted as direct evidence that steatorrhea was absent; rather, it suggests that clinically overt fat malabsorption may have been less prominent in CTD-PLE than in LDD-PLE.

An additional between-group difference was thrombotic burden. Thrombotic events were substantially more frequent in CTD-PLE, and baseline D-dimer was also higher in CTD-PLE than in LDD-PLE. Because baseline manifestations and laboratory results were defined at first admission before in-hospital treatment, these findings are unlikely to be explained by perioperative prophylactic anticoagulation in surgically treated follow-up patients. Although PLE itself may predispose to hypercoagulability due to loss of anticoagulant proteins (1), the excess thrombotic burden in CTD-PLE may also reflect autoimmune inflammation or antiphospholipid antibodies. However, provoking factors for thrombosis and antiphospholipid antibody profiles were not uniformly available in this retrospective cohort; therefore, any link to antiphospholipid syndrome should be regarded as speculative. Laboratory profiles also differed substantially. CTD-PLE showed typical autoimmune features, including anemia, hypocomplementemia, and higher ANA positivity; however, a minority of patients lacked these typical markers, complicating etiologic identification. Notably, CTD-PLE exhibited prominent hyperlipidemia, with significantly higher TC, LDL-C, and TG than LDD-PLE. An exploratory sensitivity analysis adjusting TC for baseline ALB showed that TC remained independently associated with CTD-PLE, suggesting that the lipid difference was not explained solely by the severity of hypoalbuminemia. Baseline laboratory values were defined before in-hospital treatment, which reduces confounding from therapies started after admission; however, prior outpatient glucocorticoid exposure was not uniformly captured, so some steroid-related influence on lipid levels cannot be excluded. Mechanistically, emerging evidence suggests that SLE-associated PLE may involve multiple pathways, including (i) immune complex- or complement-mediated small-vessel vasculitis with increased capillary permeability, (ii) inflammatory cytokine-mediated disruption of epithelial tight junctions, and (iii) localized intestinal lymphangiectasia secondary to bowel wall edema or increased hydrostatic pressure (8, 9). Immune-mediated mucosal injury may lead to relatively selective loss of albumin, while cholesterol-rich lipoproteins in lymph may not necessarily leak (5, 8). In contrast, in intestinal lymphangiectasia, protein- and lipid-rich lymph (e.g., chylomicrons) may leak nonselectively into the gut lumen (5). These data are compatible with different dominant leak patterns between CTD-PLE and LDD-PLE, but they do not directly prove a size-selective mucosal barrier defect.

The serum globulin and immunoglobulin profile also differed in CTD-PLE. In our cohort, CTD-PLE had significantly higher GLB and IgG/IgA/IgM levels than LDD-PLE, and some patients even exhibited hyperglobulinemia, which appears inconsistent with the classical concept of nonselective plasma protein loss in PLE (1). Possible explanations include (i) polyclonal B-cell activation in CTD leading to increased immunoglobulin synthesis (16–18), potentially offsetting gastrointestinal losses; and (ii) a “size-selective” barrier effect in inflamed mucosa, whereby larger molecules leak less efficiently than smaller proteins such as albumin. These mechanistically driven serologic differences motivated our subsequent correlation analyses.

To address cases in which CTD-PLE lacked typical immunologic markers (e.g., normal complement or negative autoantibody profiles), we explored an ROC-based diagnostic model using routine variables. A combined model incorporating age at onset, hemoglobin, and total cholesterol showed good apparent discrimination between CTD-PLE and LDD-PLE in this derivation cohort. However, because this was a single-center retrospective cohort without internal resampling or external validation, the model should be regarded as exploratory rather than ready for direct clinical implementation. Because C3 had the highest single-variable AUC and is routinely measured in many tertiary centers managing complex PLE, we also tested a *post hoc* C3-augmented sensitivity model, which further improved apparent discrimination. Nevertheless, we retained the age at onset/Hb/TC model as the primary model because it uses universally accessible variables, whereas the C3-augmented result remains exploratory and equally unvalidated. Age at onset captures epidemiologic differences (CTD-PLE often in adults, whereas congenital lymphatic anomalies or congenital heart disease-associated PLE may present in infancy or childhood). Hemoglobin may reflect differing impacts on hematopoiesis, as CTD is often complicated by anemia of chronic disease or autoimmune hemolytic anemia (8). Total cholesterol is also clinically informative: hypercholesterolemia has been identified as an independent risk factor for PLE in SLE (4). In a study by Lim et al. (19) of 14 patients with SLE-PLE, initial total cholesterol ≥240 mg/dL predicted a higher remission rate with glucocorticoids, suggesting that cholesterol may reflect the underlying immunopathophysiology and responsiveness to immunotherapy. These routine variables may provide preliminary clues to CTD-PLE when specialized immunologic testing is unavailable, but validation in independent cohorts is required.

Our correlation analyses further examined associations between baseline ALB and other indicators. In CTD-PLE, ALB was strongly negatively correlated with ESR, which is consistent with the possibility that systemic inflammation may aggravate albumin loss via intestinal barrier injury. Immune-mediated increases in intestinal microvascular permeability are considered key mechanisms for protein leakage in CTD such as SLE (16–18, 20). Systemic inflammation—through immune complexes, complement activation, and cytokines—can damage vascular endothelium and promote leakage of albumin into the intestinal lumen (21). Epithelial barrier impairment is also increasingly recognized: inflammation may activate the zonulin pathway, the only known endogenous modulator of tight junction permeability. Zonulin has been reported to be elevated in serum and stool in active SLE (18), suggesting inflammation-driven tight junction opening that enhances paracellular permeability to macromolecules such as albumin (22). Inflammatory mediators such as tumor necrosis factor-α (TNF-α) may directly disrupt tight junction structure and increase mucosal permeability (20, 23). These mediators may also downregulate protective cell-surface heparan sulfate proteoglycans (HSPGs) via reduced expression and increased shedding, which further increases susceptibility to barrier injury and amplifies protein leakage (23).

In CTD-PLE, ALB was negatively correlated with TC/LDL-C/TG as well as IgM, and positively correlated with HDL-C as well as IgG. These patterns may reflect mechanistic complexity. Immune-mediated mucosal injury may increase permeability with a size-dependent “pore” effect: smaller molecules traverse more readily, whereas larger lipoproteins and immunoglobulins may be relatively retained in circulation. Hypoalbuminemia may also stimulate hepatic lipoprotein synthesis to maintain oncotic pressure, thereby contributing to hyperlipidemia, analogous to mechanisms described in nephrotic syndrome. Consequently, smaller components may be preferentially lost (lower ALB/IgG/HDL-C), while larger components (e.g., IgM or LDL-C) may be relatively preserved or even rise, producing the observed correlation pattern. By contrast, in LDD-PLE, correlations did not show size-selective features: ALB correlated positively with GLB and lipid parameters, suggesting synchronized decreases across small and large components consistent with nonselective lymph leakage. Overall, these observations are consistent with mechanistic differences between CTD-PLE and LDD-PLE, but the analyses are correlative and hypothesis-generating; prospective studies with direct assessment of barrier and lymphatic function are needed before causal inferences can be made.

In the subset with available follow-up, CTD-PLE patients treated with glucocorticoids plus immunosuppressants and LDD-PLE patients treated surgically showed different post-treatment patterns. Specifically, 19 of 20 CTD-PLE patients achieved symptom remission, with a median albumin improvement of 9.5 g/L, whereas 23 of 43 LDD-PLE patients achieved symptom remission, with a median albumin improvement of 2.0 g/L. These observations should not be interpreted as direct evidence of prognostic or therapeutic superiority because follow-up was incomplete, durations were unequal, and treatment pathways reflected fundamentally different disease mechanisms. Previous reports suggest that SLE-associated PLE often responds well to glucocorticoids plus immunosuppressants, achieving normalization of albumin and symptom resolution within months (4, 8). In our followed CTD-PLE subset, the observed improvement after medical therapy is consistent with that general clinical experience. For surgically treated LDD-PLE, outcomes appeared more heterogeneous. This likely reflects the structural and functional complexity of lymphatic disease—particularly thoracic duct obstruction—which may require technically challenging interventions and may not always be durably corrected (1, 23–27), especially when central venous pressure is elevated (24). Prolonged lymphatic impairment may also cause irreversible structural changes or fibrosis (25, 27), contributing to chronicity and relapse. These observations underscore the importance of accurate etiologic diagnosis and mechanism-based treatment selection. Mechanistic work by Bode et al. (23) suggests that the interplay among hydrostatic pressure, inflammatory mediators, and key barrier components such as HSPGs (especially syndecan-1) may offer new therapeutic avenues: *in vitro* and animal studies showed that non-anticoagulant heparin derivatives can mitigate cytokine-induced protein loss. Future studies may explore barrier-stabilizing therapies as adjuncts to surgery or interventions, and also clarify the role of secondary inflammation in LDD-PLE progression.

We also observed that, after adjustment for baseline ALB, higher baseline serum IgA independently predicted less ALB improvement in LDD-PLE. This finding suggests that elevated IgA may reflect persistent mucosal barrier impairment or a more severe disease state. Secretory IgA (SIgA) is a key first-line mucosal defense that maintains barrier integrity and immune homeostasis via immune exclusion, microbiota regulation, and maintenance of tolerance (28–32). IgA can modulate commensal gene expression (e.g., mucus-associated functional factors) to enhance colonic homeostasis and resist inflammatory injury (33). In LDD-PLE, lymphatic stasis and associated inflammation may disrupt epithelial function, potentially impairing polymeric immunoglobulin receptor (pIgR)-mediated transport of dimeric IgA (and some IgM) into the lumen (30). Reduced pIgR expression or function would decrease luminal SIgA, weakening mucosal defense, while untransported IgA may accumulate in the lamina propria or spill into the circulation, increasing serum IgA (32, 34). Animal studies support this mechanism: pIgR knockout markedly reduces luminal IgA and increases serum IgA, indicating impaired trans-epithelial transport compromises mucosal immunity (34). Thus, in LDD-PLE, elevated baseline IgA may indirectly indicate impaired mucosal healing and defense. Alternatively, increased serum IgA could be a marker of chronic inflammation and disease severity; in inflammatory bowel disease, IgA correlates with indices such as the Crohn’s Disease Activity Index (CDAI) and CRP (35). Lymphatic stasis may impair clearance of antigens and inflammatory mediators from mucosa and surrounding tissues. In mice with impaired lymphatic function (e.g., FOXC2 downregulation), colitis is exacerbated, whereas restoration of lymphatic drainage via VEGF-C improves colitis (36). Overall, elevated IgA in LDD-PLE may reflect the extent of mucosal dysfunction or chronic disease severity. Prospective studies assessing mucosal IgA secretion and barrier markers are needed to clarify the role of IgA in LDD-PLE.

This study has limitations. As a single-center retrospective analysis, it is susceptible to information and selection bias, and the CTD-PLE subgroup was relatively small (n=30), limiting statistical power. Baseline data were defined at the first time patients met inclusion criteria in our center and may have been influenced by prior disease course or early interventions. Data completeness also varied across variables, and several potentially informative retrospective variables were not uniformly captured, including objective fat malabsorption tests, detailed stool characteristics, provoking factors for thrombosis, fibrinogen, antiphospholipid antibody panels, and standardized pre-admission glucocorticoid exposure. Comprehensive immunologic assessments were not available for all patients, which may have introduced measurement heterogeneity and limited etiologic characterization.

No formal adjustment for multiple testing was applied in exploratory secondary analyses, and the diagnostic model was neither internally resampled nor externally validated; therefore, its reported discriminative performance may be optimistic. Follow-up data were incomplete, and limited long-term follow-up restricted assessment of sustained remission and relapse.

## Conclusions

In this study, we characterized PLE in a scintigraphy-confirmed cohort and demonstrated marked differences between CTD-PLE and LDD-PLE with respect to age at onset, sex distribution, thrombotic burden, lipid profiles, and immunologic patterns. A model based on routinely available variables (age at onset, hemoglobin, and total cholesterol) showed good apparent discrimination in this derivation cohort and may assist preliminary clinical differentiation between these two major subtypes, although validation in independent cohorts remains necessary. Sensitivity analyses suggested that adding C3 may further improve discrimination, but this augmented model is also exploratory. The observed lipid, coagulation, and correlation patterns suggest that CTD-PLE and LDD-PLE may reflect different dominant leak mechanisms, while the available follow-up data indicate divergent post-treatment trajectories under medical and surgical management. Larger prospective multicenter studies are needed to confirm these findings.

## Data Availability

The raw data supporting the conclusions of this article will be made available by the authors, without undue reservation.

## Supplementary material

Supplementary Figure S1 Flowchart of patient selection, adjudication, prespecified grouping, and follow-up availability.
