# Supplementary figures and images for: Clinical characteristics and risk factors of protein-losing enteropathy: a retrospective study
Source: Front Immunol. 2026 May 8;17:1760573. doi: 10.3389/fimmu.2026.1760573 (PMC13194555; doi:10.3389/fimmu.2026.1760573)

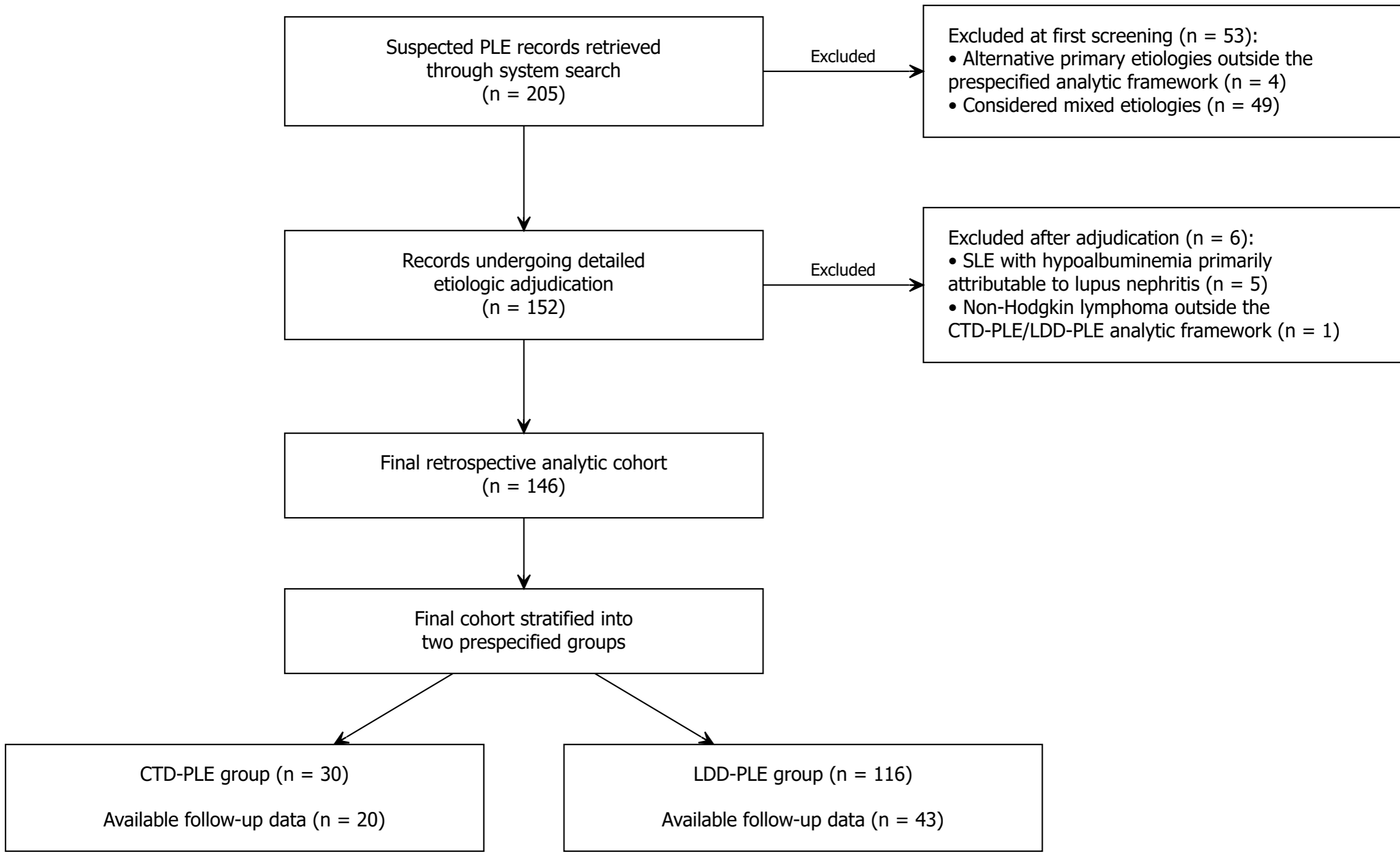

Supplement: Supplementary Figure S1 — Flowchart of patient selection, adjudication, prespecified grouping, and follow-up availability. [file Image1.pdf]
